# The application of ChatGPT in healthcare progress notes: A commentary from a clinical and research perspective

**DOI:** 10.1002/ctm2.1324

**Published:** 2023-07-02

**Authors:** Josh Nguyen, Christopher A. Pepping

**Affiliations:** ^1^ Centre for Youth Mental Health University of Melbourne Melbourne Victoria Australia; ^2^ School of Psychology and Public Health La Trobe University Melbourne Victoria Australia; ^3^ School of Applied Psychology Griffith University Brisbane Queensland Australia

**Keywords:** Artificial intelligence, ChatGPT, progress notes, prompt engineering

1

ChatGPT, powered by one of the most advanced language processing systems, gained over 100 million users in just 2 months following its release in November 2022.^1^ This unprecedented popularity is likely due to its wide range of potential applications in fields, such as engineering, education and healthcare.^2^, ^3^, ^4^ The integration of artificial intelligence (AI)—driven language models like ChatGPT has the potential to revolutionize documentation practices, streamline workflows, and ultimately lead to more efficient and patient‐centred care,^2^ though the use of such tools is not without its challenges. Here, we outline the potential benefits and pitfalls of implementing AI‐driven language models, such as ChatGPT, in the creation and management of healthcare progress notes using prompt engineering techniques. We provide recommendations for responsible and effective integration into clinical practice and priorities for future research.

Healthcare clinicians spend 35% of their time documenting patient data, and evidence suggests the length of healthcare case notes has been increasing over time.^5^ Existing innovations, such as speech recognition technology, yield no clear benefit in timesaving or documentation quality.^6^ With the ability to coherently write logical and accurate text within a few seconds, ChatGPT has the potential to reduce time spent on tasks such as preparing healthcare progress notes, and might also enhance consistency and quality. We tested the utility of ChatGPT for writing a progress note in the context of chronic pain assessment using prompt engineering, which refers to the iterative process of fine‐tuning the prompts to achieve the most desired output. These prompts included (1) the goals of the notes (e.g. write a concise and accurate progress note); (2) specific details about the patient, session and treatment plan; and (3) structure (e.g. subjective information, objective observations, assessments, plans and any interventions used). As shown in Table 1, ChatGPT could comprehend the input, which intentionally contained grammar mistakes, and elaborate on key concepts to generate a logical and relatively accurate progress note within a few seconds. In addition, when we tested the prompts without specifying the goals and suggested structure, ChatGPT generated a less precise and coherent note. This preliminary test of refining prompts for writing psychiatric notes shows the great potential of integrating ChatGPT in generating health progress notes with effective prompts.

**TABLE 1:** Prompt and output from ChatGPT.

Prompt	(Context) Write a concise and accurate health progress note. (Specific details) 35‐year female X, chronic lumbar pain (7/10) for 5 years, impact sleep and work. Third session. Med: acetaminophen 500 mg PRN—pain, amitriptyline 25 mg pain, sleep. Family history of chronic pain. Nil structure deformity or inflame. Sleep issues increase pain, stress and worry lead to sleep. Target sleep. Med review. Physio review. Appeared normal today with good eye contact and speech. Structure is subjective, objective, assessment, plan, interventions included today
Output	*Subjective*: Ms. X, a 35‐year‐old female, presents for her third therapy session. She reports chronic lumbar pain rated 7 out of 10 on a pain scale for the past 5 years. The pain significantly affects her sleep and work. Ms. X states that her pain is worsened by stress and worries, which further disrupt her sleep. She has a family history of chronic pain. No structural deformities or signs of inflammation are noted. *Objective*: Ms. X is currently taking acetaminophen 500 mg PRN for pain relief and amitriptyline 25 mg for pain and sleep. Today, she appeared normal with good eye contact and clear speech. *Assessment*: Ms. X continues to experience chronic lumbar pain, rating it as severe and reporting its negative impact on her sleep and work. Addressing sleep issues is a priority as they contribute to pain exacerbation. No structural abnormalities or signs of inflammation are observed. *Plan*: Medication review: Evaluate the efficacy of the current pain management regimen, including acetaminophen and amitriptyline. Adjustments may be considered based on response and side effects. Physiotherapy review: Refer Ms. X for a comprehensive evaluation by a physiotherapist to address musculoskeletal imbalances, provide targeted exercises and improve functional capacity. Sleep improvement: Focus on enhancing sleep quality through sleep hygiene practices, relaxation techniques and stress reduction strategies. Regular monitoring: Schedule follow‐up sessions to assess progress, evaluate pain levels and make any necessary modifications to the treatment plan. *Interventions*: ‐ Reviewed medication use and discussed potential adjustments. ‐ Recommended a physiotherapy evaluation for targeted pain management and functional improvement. ‐ Introduced sleep hygiene practices and relaxation techniques to promote better sleep. ‐ Scheduled the next session for ongoing treatment and progress evaluation. Note: Ms. X presents with chronic lumbar pain that significantly impacts her sleep and work. The treatment plan emphasizes addressing sleep disturbances as a key factor in pain management. Medication review, physiotherapy evaluation and interventions targeting sleep improvement will be implemented to enhance Ms. X's overall well‐being. Regular follow‐up sessions will allow for monitoring progress and adjusting the treatment plan as needed.

Despite these strengths, it is important that healthcare clinicians and organizations are aware of the challenges and limitations of using ChatGPT for healthcare progress notes. First, the accuracy and reliability of ChatGPT‐generated content are contingent upon the quality of input data and the effectiveness of prompting techniques. If the input data contain errors or inconsistencies, ChatGPT is likely to produce inaccurate or misleading information, which could have serious implications for patient care. Second, ChatGPT is trained using data on the Internet, which could potentially have inherent biases (e.g. racial, gender and/or cultural biases).^7^ At this early stage, providers must remain vigilant to ensure that the use of AI does not compromise the quality of care, lead to an overreliance on technology or perpetuate biases present in training data. We argue that AI tools should be used as an assistance tool rather than a replacement for human input; the expertise of clinicians remains essential for evaluating the quality of auto‐generated progress notes.

As with any digital technology in healthcare, ensuring the privacy and security of sensitive patient information is paramount. Sensitive information submitted to ChatGPT can have severe consequences.^8^ Healthcare organizations that aim to implement models like ChatGPT would need comprehensive guidelines for using such tools in handling patient data and implement measures to secure the privacy of patient data (e.g. anonymizing identifiable data, encryption, compliance with national healthcare regulations, etc.). Some recently developed AI‐powered software claims to harness the cutting‐edge capabilities of ChatGPT while meeting the requirements for data security and privacy,^9^, ^10^ though the effectiveness of these software tools in clinical settings requires rigorous validation and quality control processes. There is a need for interdisciplinary collaboration between AI developers, healthcare clinicians, policymakers and data security experts.

In conclusion, the integration of ChatGPT with effective prompting techniques holds great potential for streamlining healthcare documentation, yet it must be approached carefully to manage ethical and practical challenges, and to prevent harm. Adoption of this technology may yield significant benefits in healthcare documentation, which has the potential to improve clinician productivity and patient care. Guidelines for the use of AI tools in patient data documentation will be an important next step, and research efforts are needed to investigate the effectiveness of these tools compared to existing methods.
